# Wearable airbag technology and machine learned models to mitigate falls after stroke

**DOI:** 10.1186/s12984-022-01040-4

**Published:** 2022-06-17

**Authors:** Olivia K. Botonis, Yaar Harari, Kyle R. Embry, Chaithanya K. Mummidisetty, David Riopelle, Matt Giffhorn, Mark V. Albert, Vallery Heike, Arun Jayaraman

**Affiliations:** 1 Max Nader Rehabilitation Technologies and Outcomes Lab, Shirley Ryan AbilityLab, Chicago, IL USA; 2 Department of Physical Medicine and Rehabilitation, Northwestern University, Chicago, IL USA; 3 Northwestern University Feinberg School of Medicine, Chicago, IL USA; 4 Department of Computer Science and Engineering, Department of Biomedical Engineering, University of North Texas, Denton, TX USA; 5 Department of BioMechanical Engineering, Delft University of Technology, Delft, The Netherlands; 6 Department of Rehabilitation Medicine, Erasmus MC, Rotterdam, The Netherlands

**Keywords:** Pre-impact fall detection, Fall mitigation, Machine-learning, Wearable sensors, Stroke, Injury prevention, Rehabilitation

## Abstract

**Background:**

Falls are a common complication experienced after a stroke and can cause serious detriments to physical health and social mobility, necessitating a dire need for intervention. Among recent advancements, wearable airbag technology has been designed to detect and mitigate fall impact. However, these devices have not been designed nor validated for the stroke population and thus, may inadequately detect falls in individuals with stroke-related motor impairments. To address this gap, we investigated whether population-specific training data and modeling parameters are required to pre-detect falls in a chronic stroke population.

**Methods:**

We collected data from a wearable airbag’s inertial measurement units (IMUs) from individuals with (*n* = 20 stroke) and without (*n* = 15 control) history of stroke while performing a series of falls (842 falls total) and non-falls (961 non-falls total) in a laboratory setting. A leave-one-subject-out crossvalidation was used to compare the performance of two identical machine learned models (adaptive boosting classifier) trained on cohort-dependent data (control or stroke) to pre-detect falls in the stroke cohort.

**Results:**

The average performance of the model trained on stroke data (recall = 0.905, precision = 0.900) had statistically significantly better recall (*P* = 0.0035) than the model trained on control data (recall = 0.800, precision = 0.944), while precision was not statistically significantly different. Stratifying models trained on specific fall types revealed differences in pre-detecting anterior–posterior (AP) falls (stroke-trained model’s F_1_-score was 35% higher, *P* = 0.019). Using activities of daily living as non-falls training data (compared to near-falls) significantly increased the AUC (Area under the receiver operating characteristic) for classifying AP falls for both models (*P* < 0.04). Preliminary analysis suggests that users with more severe stroke impairments benefit further from a stroke-trained model. The optimal lead time (time interval pre-impact to detect falls) differed between control- and stroke-trained models.

**Conclusions:**

These results demonstrate the importance of population sensitivity, non-falls data, and optimal lead time for machine learned pre-impact fall detection specific to stroke. Existing fall mitigation technologies should be challenged to include data of neurologically impaired individuals in model development to adequately detect falls in other high fall risk populations.

*Trial registration* https://clinicaltrials.gov/ct2/show/NCT05076565; Unique Identifier: NCT05076565. Retrospectively registered on 13 October 2021

**Supplementary Information:**

The online version contains supplementary material available at 10.1186/s12984-022-01040-4.

## Background

Every year, approximately 13 million individuals around the world experience a stroke [1, 2]. Falls are one of the most common medical complications experienced by individuals after a stroke, reported in up to 65% of the stroke population during hospitalization and up to 75% in the community [3, 4]. Individuals who have experienced a stroke are at an increased vulnerability for falling, related to common correlates of high fall risk in this population such as impaired mobility, medication use, and cognitive impairment [5]. Falling after a stroke can have serious consequences. There are a high incidence of severe physical injuries, including fractures, soft tissue and head injuries, and at worst, death [6, 7]. Psychologically, individuals often develop a fear of falling, leading to reduced mobility, increased social isolation, and significant reduction in quality of life [8, 9]. Financially speaking, fall related injuries constitute a burden on healthcare systems through prolonged use of services and incurred high healthcare costs [10–12]. Despite evidence that multifactorial rehabilitation approaches such as improving strength, balance, and visual impairments can reduce fall incidence in the older adult population [13], a recent Cochrane review concluded that there is little to no evidence of interventions that can prevent falls from occurring in individuals experiencing falls after stroke [14]. Therefore, individuals who suffer from mobility deficits after a stroke continue to experience falls, frequently and repeatedly. Without a way to prevent these falls from occurring, there is a compelling need to develop methods and tools to detect these falls before impact with the ground and reduce the associated consequences.

One conventional solution to achieve some degree of fall impact mitigation is wearing padded hip protectors in or underneath clothing, yet their significance in reducing fractures and associated injuries is limited and their current usage in the community is insignificant (likely due to discomfort and poor compliance) [15, 16]. A more novel and recent fall impact mitigation approach to address these concerns is wearable airbag technology [17–20]. These devices generally include three design components: (1) at least one sensor, such as an inertial measurement unit (IMU), to record user motion; (2) a computational model that processes the sensor signals to pre-detect fall impact; and (3) an inflatable airbag mechanism that deploys upon detection of a fall, to mitigate contact forces with the ground.

Despite continued development, wearable airbag technologies are currently only developed with the non-neurologically impaired older adult population in mind. Furthermore, the internal fall impact detection algorithms are often developed on data from young participants [21–25]. The algorithms have neither been specifically designed nor validated for detecting falls of individuals presenting with stroke-related motor impairments. A presumption is that the design and computational models should seamlessly transfer to users in the stroke population. However, the underlying pathophysiology of a stroke, fundamentally related to the cerebrovascular territory that is compromised in the brain, may manifest with alterations in movement kinematics and a loss of ability to control movements. These characteristic changes in movement have been observed and quantified in existing literature [26–28] and may translate to observed and measurable differences leading up to or during falls [29–33]. For example, earlier studies have analyzed and compared falls between older able-bodied and stroke individuals, and found significantly different motor responses including postural stability, trunk control, fall velocity, and timely step compensation [29, 31–33]. Furthermore, Dusane et al. found that within a stroke population, kinematic responses differed depending on the side of the body which a fall was initiated on (paretic vs. non-paretic) [34]. Given falls in stroke may have distinct kinematic profiles, current fall mitigation technology developed on data of generally healthy individuals might not be sufficiently sensitive or specific to detect falls in individuals who have experienced a stroke. Such inaccuracy could result in failure to deploy the airbag during a fall or cause unnecessary airbag deployments (i.e. false positives) and consequently, may lead to poor user engagement.

To address these issues, we suggest that systematic fall detection models should consider incorporating training data of individuals specific to the intended user population. Using machine learning to tune movement recognition algorithms to unique movements of particular mobility-impaired populations has been demonstrated in various applications for Parkinson’s disease [35]*,* incomplete spinal cord injury [36, 37], and stroke [38], yet has not been applied to pre-impact fall detection in stroke populations. Thus, this paper presents considerations for a sensor-based, machine learned wearable airbag system to demonstrate the importance of pre-impact fall detection models specific to stroke-related movement impairments. We hypothesize that for fall detection in the stroke population, a pre-impact fall detection model trained on data from a stroke population would perform better than models trained on data from a control population. In other words, failure to train a model on fall movements specific to individuals with a history of stroke will result in decreased pre-impact detection performance for users of the stroke population. Furthermore, we explore secondary considerations for model development, including dataset activity composition, severity of gait impairments across users, and lead time parameter tuning.

## Methods

### Device concept and prototype

**Fig. 1:**
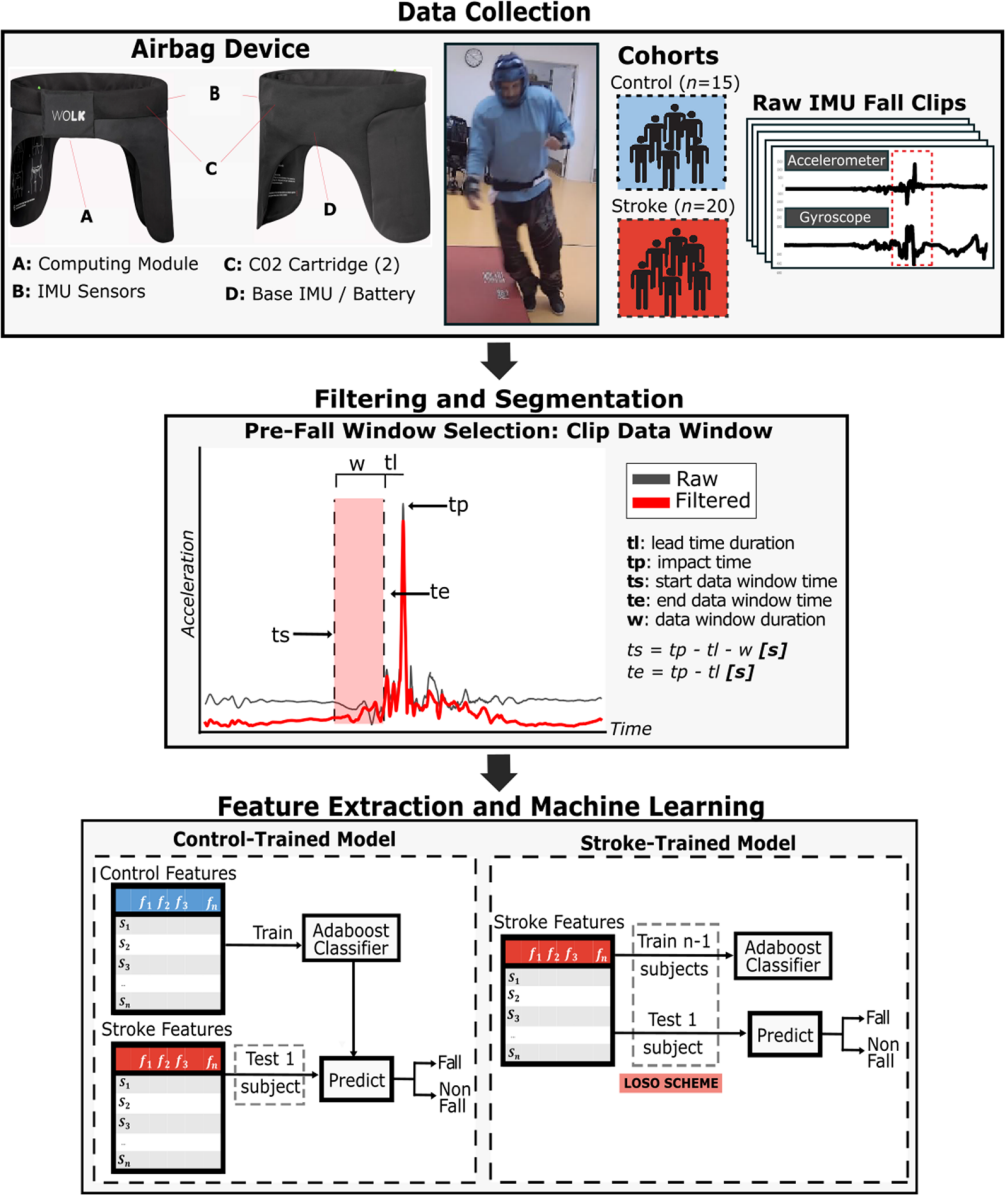
**Airbag Device and Model Pipeline**. Sequence of steps in processing and developing the fall prediction models. Kinematic data were collected from individuals with a history of stroke (*n* = 20) and individuals who had not experienced a stroke (i.e. control, *n* = 15) while wearing an IMU airbag device. Raw IMU accelerometer and gyroscope signals were filtered and segmented for the pre-impact fall data window, ending at the detected impact time minus the selected lead time duration. Statistical features were extracted from the pre-impact fall data window and labeled according to cohort membership (control or stroke) and activity type (fall or non-fall, fall type). All features were used as input to two models, each model trained on either control or stroke data. The control-trained model was trained on all control features, and each subject of the stroke cohort was tested iteratively. In the stroke-trained model, a single subject was iteratively held out for testing while the remaining subjects were used for model training (leave-one-subject-out cross-validation scheme). The dashed grey rectangle signifies an iterative selection process of each held out test subject. Performance metrics to pre-impact detect falls in stroke were compared between the two exclusive cohort trained models

Data were recorded via the Wolk Hip Airbag (Wolk De Heupairbag; Wolk Company, Netherlands), a commercial airbag system (Fig. 1). Wolk Airbag is a Conformitè Europëenne (CE) marked and Federal Communications Commission (FCC) approved smart system designed to mitigate falls in the healthy older adult population. The device is a lightweight, battery-powered belt available in four sizes designed to be worn underneath clothing. The device includes three fixed IMU sensors (one on the side of each hip and one on the lower back, in line with the L3 vertebrae) and an onboard computing unit. Each IMU sensor contains an accelerometer (range ± 16 g) and gyroscope sensor (range ± 2000 deg/s) collecting data in all three axes (x, y, and z) at a sampling rate of 500 Hz. The device has built-in models for pre- and post-impact fall detection, which send a command signal for deployment to the two embedded ${\mathrm{CO}}_{2}$ cartridges in the event a fall impact is pre-detected. For this study, a modified version of the Wolk airbag was utilized. The internal circuitry had been altered to incorporate data logging capabilities to an external SD card to store the raw IMU data, and the deployment signal was inhibited to prevent cartridge activation during the supervised falls data collection.

### Study design and data collection

Stroke cohort eligibility criteria included individuals within the age range of 18–85, diagnosis of stroke at least 6 months ago, and the ability to sit unsupported and walk independently (at least with an assistive device). The control cohort were recruited from the same age range with no serious conditions, injuries, or history of back pain. Exclusion criteria for both cohorts included persons on anti-coagulants, severe osteoporosis, pregnant women, cognitive deficits, and a comorbidity that interferes with the efficacy of the study or increases minimal risk of injury. All participants signed informed consent before study participation, which was approved by the Northwestern University Institutional Review Board (NUIRB, IL, USA). All study procedures were carried out in accordance with the standards listed in the Declaration of Helsinki 1964. This study is a registered clinical trial on ClinicalTrials.gov (registry number NCT05076565) with the National Library of Medicine (NLM) at the National Institutes of Health (NIH).

Participants wore the airbag device and protective gear (e.g., helmet, neck/knee/elbow guards, padded shorts) while performing falls and non-falls onto a padded mat under supervision. For the purposes of this study, a fall was defined as an event in which the participant loses balance and results in a terminal position on the ground (e.g., slips, trips, and falls from chair) [39]. Within falls, we defined subcategories for the directionality of the fall, namely in the lateral direction (i.e., lateral fall) and anterior–posterior direction (i.e., AP fall). Participants were instructed to respond to loss of balance using any natural technique, such as using an arm to catch themselves or taking multiple steps, to encourage realistic behavior. Non-falls were performed and stratified for low or high movement complexity, in this case related to loss of balance. Activities of daily living (ADLs) are lower complexity non-fall movements which do not include loss of balance (e.g., walking, sitting, lying, sit-to-stands, jumping), and are often used to discriminate against fall events for fall detection technology. On the higher complexity end, near-falls are non-fall movements in which the participant loses balance but recovers without hitting the ground, such as recovery from slips, trips, and lateral perturbations. The order of the event types (i.e., falls, non-falls) and their subcategories (e.g., trips, slips, near-falls, ADLs) were randomized between participants. Each event was observed and labeled by the research team, encoding the type of fall activity and its context. Video recordings of all activities were collected for fall activity confirmation, post-session processing, and data analysis. All events were performed at least twice to capture intra-subject variability. Participants did not use assistive mobility devices during the session with the exception of orthoses if necessary.

### Development of a fall detection model for individuals with stroke-related impairments

The collected raw IMU data and event times were provided as input to an automated custom Python program (Fig. 1). Accelerometer data were filtered by a fourth-order band pass Butterworth filter (0.1–50 Hz) to remove high frequency noise. The pre-impact data window was extracted based on the fall impact and the acceptable intervention time, which is critically constrained by the device lead time (i.e., the time prior to impact by which the algorithm must decide to deploy the airbag for successful intervention) [23]. The minimal lead time for the device in use is 75 ms. Thus, for each fall event, the program automatically selected an evaluation data window (250 frames) ending 75 ms before the identified fall impact, i.e., the last possible moment to classify the fall (Fig. 1). Non-fall data were randomly evaluated within the latter half of the event time region to capture near-fall event features in close proximity to the user’s maximal instability for the task. Any activities with missing or defective data (due to occasional hardware damage of a cable or connection during recording) were flagged and removed from analyses. The following features were engineered for each axis of the acceleration and angular velocity from the IMU signals: min, median, max, interquartile range, standard deviation, skew, and kurtosis (see Additional file 1: Table S1) [40].

The calculated features were used as input to develop a machine learning classifier for pre-impact fall detection based on an adaptive boosting algorithm [41]. Gradient boosting algorithms, such as AdaBoost, have been noted for superior fall detection performance as compared to other methods including k-Nearest Neighbor (KNN), Support Vector Machine (SVM), and Random Forest (RF) [42]. The AdaBoost classifier is a multiple decision tree classifier that, in sequence, chooses a selective weight by which the average contributes to the final prediction of the test data set. Two identical AdaBoost models (50 estimators, learning rate = 1) were developed to detect falls of individuals with stroke-related impairments. Each model was exclusively trained on data from a homogeneous population, namely, (i) a model trained only on data of non-stroke participants (the “control-trained” model), and (ii) a model trained only on data of post-stroke participants (the “stroke-trained” model). A “leave-one-subject-out” (LOSO) modeling scheme was used to structure train and test sets in the stroke-trained model. The control-trained model was trained on data of all control subjects and iteratively tested on each stroke cohort participant (Fig. 1). Both models utilized identical processing techniques and features for iterative model development and testing. Events were predicted and labeled as falls or non-falls.

One of the key factors in developing a generalized and reliable fall detection model is the type of data used for the non-fall events. In much of the existing research, falls are improperly classified against static ADLs only, which often yields inflated and overly optimistic fall detection performance. Real life applications require classification of more complex motions, such as near-fall events. In order to demonstrate the importance of non-falls diversity, fall detection models that were trained and tested using exclusively ADLs or near-falls as the non-fall events were compared. Additionally, as an exploratory analysis, we investigated whether models developed to detect falls in post-stroke individuals should include training data not only of falls from the stroke population but furthermore, falls of individuals with varying levels of stroke-related impairment severity. Models which are not adequately trained on movements of stroke individuals with severe motor impairments may result in a decreased pre-impact fall detection performance for severely impaired stroke users. To preliminarily assess the sensitivity of the models to severity representation, subjects from the available stroke cohort who displayed indicators of severe gait impairments during a standardized gait assessment were identified and collectively labeled as “unstable ambulators” (see Additional file 2: Table S2). A compilation of test sets was constructed using a “leave-one-group-out” (LOGO) modeling scheme, each group a subset of five stroke cohort subjects labeled by quantity of unstable ambulators being tested on (see Additional file 3: Figure S1). Iterative model evaluation was performed for each test group in both the control- and stroke-trained models. Please see the Additional file 5 for detailed information on this analysis.

Lastly, model performance was investigated for varied lead times to determine the optimal lead time which maximizes model performance for each population-specific model. Lead time, ${t}_{l}$, is defined as the time before the impact by which the fall must be detected [23]. Instead of viewing this lead time parameter as a constraint, we rather investigate and define an optimal lead time,${{t}_{l}}^{*}$, as the duration of time before a fall impact, ${t}_{p}$, to end the data evaluation window, ${t}_{e}$, resulting in a maximized fall-classification performance. The start, ${t}_{s}$, and end, ${t}_{e}$, time of the data evaluation window for different lead times, ${t}_{l}$, are defined as the following, where $w$ is the duration of the data window (250 frames or 500 ms):

$${t}_{s}= {t}_{p}- {t}_{l}-w$$

$${t}_{e}= {t}_{p}- {t}_{l}$$

For all analyses, the evaluated metrics on model performance were recall, precision, F_1_-score, receiver operating characteristic curve (ROC), and area under the curve (AUC). In this study, recall is defined as the percentage of true falls detected out of all true falls performed, and precision is defined as the percentage of true falls detected out of all the events predicted as falls. The F_1_-score considers both the recall and precision scores to measure overall performance, given by the following equation:

$$F1=2 \cdot \frac{recall \cdot precision}{recall+precision}$$

While performance metrics are calculated for every model iteration (i.e. each train and test split), the average and standard deviation of model performance are reported to convey overall model performance and reduce complexity for between-model comparisons.

Two-tailed paired *t* tests were performed to determine the relative performance of two models using different training data. Both models were used to make predictions for an identical test set, from which average recall, precision, F_1_-score, and AUC metrics were calculated. Each of these performance metrics were recorded per test iteration in both models, resulting in paired distributions of average performance metrics. For most tests, basic statistical significance is reported if *P* < 0.05. For some instances where repeated *t* tests were used, significance after accommodating for the family-wise error rate is also reported using the Holm-Bonferroni method [43]. The Holm-Bonferroni method proceeds as follows: for each of the *m* repeated *t* tests, sort their corresponding *P* values from lowest to highest (*P*_1_, *P*_2_,…, *P*_*m*_). Starting with the lowest *P* value, *P*_1_, check if:

$${P}_{k}<\frac{\alpha }{m+1-k},$$

where $\alpha$ is the desired significance level ($\alpha =0.05$ throughout this paper). While checking ascending values of ${P}_{k}$= *P*_1_, *P*_2_,…, *P*_*m*_, determine the first ${P}_{k}$ such that the above inequality is satisfied. All subsequent values of *P (*${P}_{k}, {P}_{k+1},\dots , {P}_{m}$*)* are considered significant. This procedure ensures that the family-wise error rate does not exceed $\alpha$, and is less overly conservative than the popular Dunn-Bonferroni method.

## Results

### Fall mitigation model performance: control- vs stroke-trained models

A summary of participant demographics and session-related characteristics are given in Table 1. In total, 842 falls (610 lateral falls and 232 AP falls) and 961 non-falls (562 ADLs and 399 near-falls) were performed in the experiment. The control cohort constitutes a greater number of recorded movement samples within all subcategories of falls and non-falls, with the exception of lateral falls. In total, 126 features per fall sample were included in the feature matrix, provided as input to the training stage of each model (see Additional file 4: Fig. S2 for an illustration of model feature importance).

**Table 1:** Participant demographic and session information

Characteristics	Unit	Stroke (n = 20)	Controls (n = 15)
Mean Age (std)	years	53 (13)	40 (16)
Sex	–	10 M / 10 F	8 M / 7 F
Mean Height (std)	cm	168.3 (12.0)	170.7 (12.8)
Mean Weight (std)	kg	80.6 (21.5)	80.3 (12.3)
Mean BMI (std)	kg/m^2^	28.1 (5.15)	27.9 (5.97)
Time Since Stroke (std)	years	7.47 (4.58)	–
Mean Gait Speed (std)	m/s	0.88 (0.29)	1.22 (0.19)
Falls Count (Lateral/AP)	–	439 (327/112)	403 (283/120)
Non-Falls Count (ADL/Near)	–	415 (259/156)	546 (303/243)

**Fig. 2:**
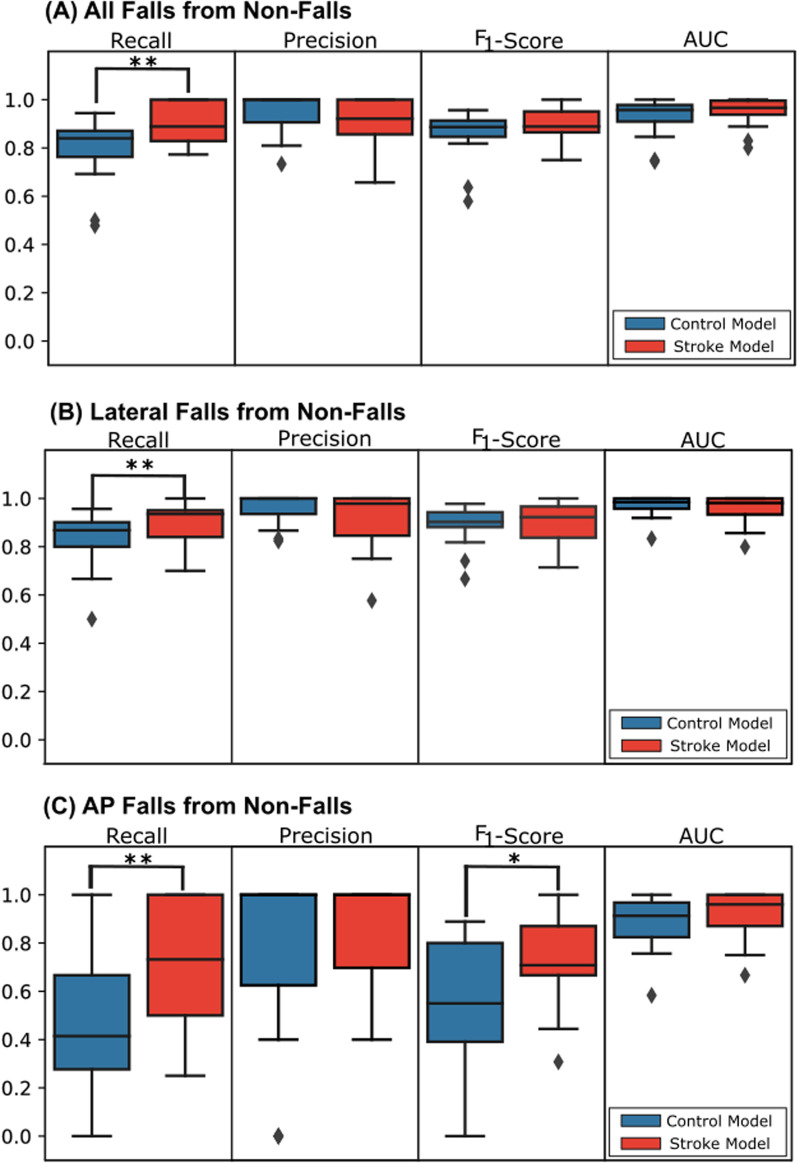
**Pre-impact fall detection performance of post-stroke individuals’ falls tested on control- and stroke-trained models.** Average performance metrics of recall, precision, F_1_-score, and AUC for pre-impact fall detection of post-stroke individuals’ falls using an AdaBoost Classifier trained on control (blue) or stroke (red) cohort data. Each model is stratified and compared across different fall types: (**A)** all falls, (**B)** lateral falls, (**C)** AP falls using paired *t* tests. Tests that resulted in a statistically significant difference (as defined by *P* < 0.05) are indicated by a single *. Tests that are significant after compensating for the family-wise error rate of using repeated *t* tests for all three fall types, as defined by the Holm-Bonferroni method, are marked with **

Cohort-dependent models were compared to detect falls of individuals with stroke-related impairments. For training on all categories of falls and non-falls, the stroke-trained model resulted in higher average recall while the control-trained model resulted in higher average precision (Fig. 2). However, the difference in recall was statistically significant (*P* = 0.0035), while the difference in precision was not. When broken down into specific fall types (i.e. lateral falls and AP falls) classified against non-fall activities, the stroke-trained model had statistically higher recall for both lateral (*P* = 0.028) and AP falls (*P* = 0.0036). The control model had 5.7% higher precision for lateral falls, while the stroke-trained model had 10.7% higher precision for AP falls, though neither difference was statistically significant.

For lateral falls, the stroke-trained model and control-trained model had approximately equal AUC and F_1_-scores, with less than 3.5% change and no statistical difference. For AP falls, the stroke-trained model had 3.5% higher AUC and 35% higher F_1_-score (*P* = 0.019).

### Comparison of data types used for fall and non-fall events

In addition to evaluating performance for directionally distinct fall types, non-falls of different movement complexities (i.e., ADLs, near-falls) were assessed (Table 2). Table 2 shows the results of paired *t* tests that were used to compare the distribution of each performance metric (recall, precision, F_1_-score, and AUC) between the validation set of stroke-trained and control-trained models. Tests that resulted in a statistically significant value (as defined by *P* < 0.05) are indicated by a single *. Tests that are significant, even after compensating for the family-wise error rate of using repeated *t* tests for all three fall types (all falls, lateral falls, AP falls), are marked with **. Using ADLs as non-fall data resulted in generally higher model performance compared to using near-falls as non-fall data. This effect was most pronounced in the control model for AP falls, where using ADLs as non-fall data resulted in statistically significantly higher recall (*P* = 0.0065), AUC (*P* = 0.042), and F_1_-score (*P* = 0.037). The difference was less pronounced for the stroke-trained model, which only has statistically significantly higher AUC when using ADL data, both when analyzing AP falls (*P* = 0.033) or the group of all falls (*P* = 0.021), but not for the group of lateral falls alone.

**Table 2:** Effect of non-fall type (ADLs or near-falls) on pre-impact fall detection for stroke- and control-trained models

	Recall		Precision		${\mathbf{F}}_{1}$-Score		AUC	
	*M*	*SD*	*M*	*SD*	*M*	*SD*	*M*	*SD*
*Stroke-trained model*								
**All falls**								
ADL	0.95	0.07	0.94	0.06	0.94	0.05	0.97	0.04
Near-fall	0.93	0.07	0.92	0.08	0.92	0.06	0.92	0.08
*P*	**0.53**		**0.3**		**0.27**		0.02*	
**Lateral falls**								
ADL	0.93	0.08	0.96	0.06	0.94	0.06	0.97	0.05
Near-Fall	0.92	0.08	0.93	0.10	0.92	0.07	0.95	0.05
*P*	**0.73**		**0.28**		**0.33**		**0.19**	
**AP Falls**								
ADL	0.79	0.23	0.84	0.20	0.79	0.19	0.97	0.05
Near-Fall	0.80	0.23	0.89	0.19	0.82	0.19	0.88	0.16
*P*	**0.81**		**0.44**		**0.65**		0.03*	
*Control-trained model*								
**All falls**								
ADL	0.88	0.12	0.92	0.07	0.89	0.08	0.94	0.08
Near-fall	0.85	0.10	0.96	0.06	0.90	0.08	0.91	0.13
*P*	**0.42**		**0.07**		**0.85**		**0.37**	
**Lateral falls**								
ADL	0.91	0.13	0.97	0.04	0.94	0.08	0.98	0.04
Near-fall	0.89	0.07	0.95	0.08	0.92	0.06	0.96	0.05
*P*	**0.56**		**0.25**		**0.45**		**0.15**	
**AP falls**								
ADL	0.75	0.22	0.82	0.25	0.76	0.21	0.94	0.07
Near-Fall	0.49	0.34	0.76	0.40	0.57	0.34	0.86	0.15
***P***	0.01**		**0.58**		0.04*		0.04*	

Average recall, precision, ${\mathrm{F}}_{1}$-score, and AUC reported for stroke-trained (top) and control-trained (bottom) models to compare the effect of non-fall type (ADL, near-fall) on model performance for each category of fall types (all, lateral, AP). For each fall type, *P* values are reported to compare the significance when using ADLs versus near-falls for the non-falls type in the model. Tests that resulted in a statistically significant value (as defined by *P* < 0.05) are indicated by a single *. Tests that are significant after compensating for the family-wise error rate of using repeated *t* tests for all three fall types are marked with **

### Model performance for a spectrum of stroke-related impairment

**Fig. 3:**
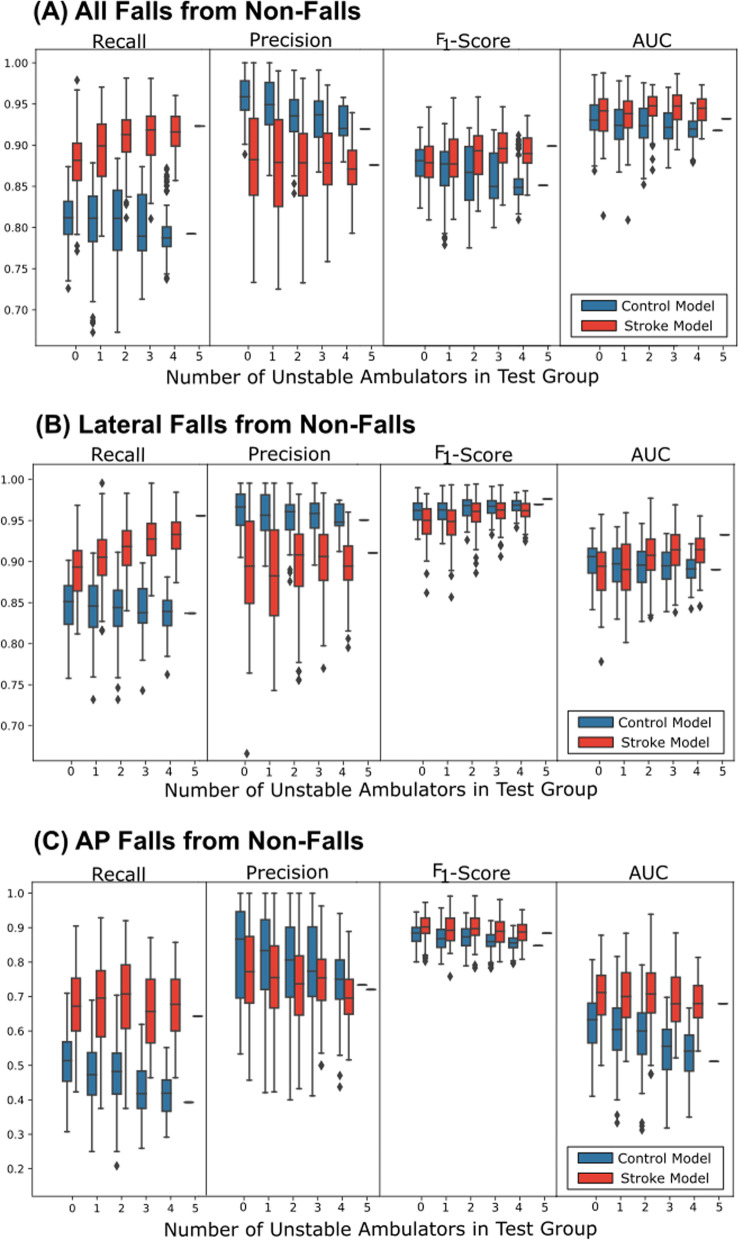
**The effect of an increasing quantity of test subjects with severe stroke-related impairments on pre fall detection.** Average recall, precision, F_1_-score, and AUC are displayed for unique, randomly selected subgroups of stroke individuals (*n* = 5 per subgroup) tested on both control- and stroke-trained models. Performance is stratified by the quantity of stroke individuals with severe stroke-related impairments (i.e. unstable ambulators) included in the test set. For zero to three unstable ambulators in a test set, 100 unique subgroups were input into both models. For four or five unstable ambulators in a test set, the maximum number of unique possible subgroups were used, i.e. 75 and 1 subgroup(s), respectively. Visual trends are displayed to demonstrate how an increase in the severity of the test group impacts model performance for each subcategory of fall types: (**A)** all falls, (**B)** lateral falls, (**C)** AP falls. Notably, control-trained model performance generally declines with an increase in unstable ambulators tested upon, while stroke-trained models are unaffected or even result in an improved performance with an increased number of unstable ambulators tested upon

The above models evaluated training data based on binary presence or absence of stroke, yet within the stroke population there is a spectrum of severity levels related to mobility and fall risk. Five stroke cohort subjects were identified and labeled as unstable ambulators (i.e., displaying some form of gait instability) from the available dataset (see Additional file 5). The distribution of model performance is demonstrated below in Fig. 3, separated and ordered by an increasing quantity of unstable ambulators in the test set. Consistent with previous findings, fall detection recall and precision face a general tradeoff in performance between the control- and stroke-trained models. However, these results suggest fall performance may be correlated to the quantity of unstable ambulators within the test set. In general, an increase in the tested quantity of unstable ambulators decreased the recall and precision of the control model, while the stroke-trained model was more or less unaffected with an increase in the quantity of unstable ambulators tested on. Accordingly, this led to a larger gap between the recall and a narrowed gap in precision between the two models, suggesting differences in the trained models’ ability to predict falls of more severely impaired stroke individuals.

### Model performance for different lead times

**Fig. 4:**
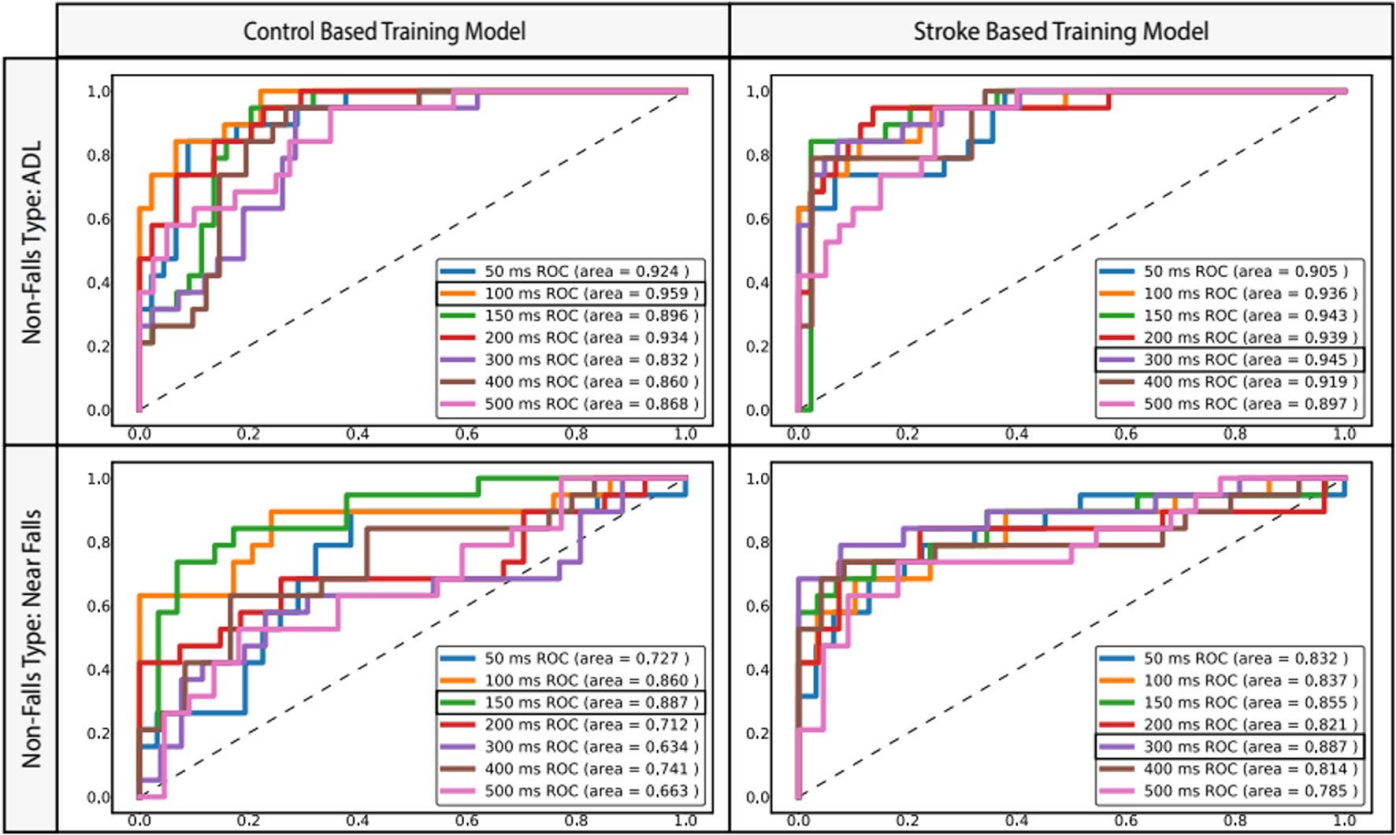
**Pre-impact fall detection optimal lead time dependent on model and non-fall type.** An AdaBoost Classifier trained on control or stroke data were used to classify AP falls against ADLs or near-falls for a test subgroup of unstable ambulatory subjects (*n* = 5). AUC-ROC curves are displayed for varied lead times (50, 100, 150, 200, 300, 400, 500 ms) for classifying AP falls from ADLs in the control-trained model (**A**) and stroke-trained model (**B**), and from near-falls in the control-trained model (**C**) and stroke-trained model (**D**). The control-trained model performs similarly to the stroke-trained model’s maximal performance with a ~ 50% lead time reduction

To test the effect of the lead time on the model’s performance, we compared the AUC-ROC curves for both models to classify AP falls against each non-fall subtype separately (ADLs and near-falls) with varied lead times (50, 100, 150, 200, 300, 400, 500 ms). The results show that for the events recorded in this study, the lead times resulting in the best performance (i.e., highest AUC values) differed between control- and stroke-trained models (Fig. 4). For classifying the unstable ambulators’ AP falls against ADLs, the control-trained model performed best at 100 ms lead time (95.9% AUC) while the stroke-trained model performed best within 150 ms–300 ms lead time (94.5% AUC). This lead time difference maintained a similar ratio when classifying against near-falls, with peak AUC scores of 88.7% in both models at lead times of 150 ms for the control-trained model and 300 ms for the stroke-trained model. As shown for both models, the lowest lead time (50 ms) did not result in the highest-performing lead time and in addition, the decrease or increase in AUC was not linear.

## Discussion

Here we present for the first time research on a wearable airbag technology specific to detecting falls in individuals that have experienced a stroke, a population at high fall risk with minimal evidence for reliable fall intervention strategies [14]. In this work, we investigate the importance of population-specific models to detect falls in stroke by purposefully altering model-dependent training data sets and analyzing the consequent effects on performance. The resulting model comparisons support our primary hypothesis, i.e. that stroke-specific training data improves pre-impact fall detection in stroke, and demonstrate the influence of activity composition, impairment severity, and lead time on stroke-specific model performance. Incorporating a population-specific model into fall mitigation technology tailored to the motion of ambulatory individuals after stroke could help to reliably mitigate the impact of falls and their related consequences in the stroke population.

This work supports recent initiatives for “data-centric artificial intelligence”, encouraging the formulation of better datasets (specific to an end model’s intended goal) rather than overdeveloping algorithms around poor data [44]. While studies have presented wearable airbag technologies for fall mitigation, their target population was non-neurologically impaired and otherwise healthy older adults and thus, detection models were developed and validated based on data of healthy individuals. To the best of our knowledge, the fall mitigation model presented in the current study is the first which intentionally encompasses movement data of stroke survivors in the training set of the detection model. Results demonstrate that the model performance for detecting falls in individuals after stroke is improved (increased recall and F_1_-score) when the model is trained using movement data of individuals after stroke, as opposed to data of healthy individuals. In this, a trend in the recall-precision tradeoff was observed, namely, stroke-trained models performed with an averaged higher recall while control-trained models performed with an averaged higher precision (though the precision difference was not statistically significant). The resulting difference in recall between the control- and stroke-trained models suggests that current fall mitigation technology developed on data of able-bodied individuals could be further improved to better protect individuals with neuromotor impairments such as stroke, supporting our hypothesis to consider stroke-specific data in the model development.

Development and validation of generalized fall detection models requires not only the aforementioned inclusion of data from the intended user population, but also a diverse and complex set of non-fall data [45, 46]. The results demonstrate that using only ADLs as non-fall data may result in inflated model performance compared to the models’ true ability to detect falls in everyday life [47, 48]. This was especially apparent for the condition in classifying AP falls from near-falls rather than ADLs, which significantly affected the performance of both control- and stroke-trained models.

For the model presented in this work, selection of the lead time has a meaningful impact on model training and evaluation. Using the shortest possible lead time may not result in the best window of data to assess the model’s true ability to detect falls. The results of this study convey that the control-trained models utilized data closer to the time of impact (shorter lead time) than the stroke-trained models to maximize performance. This could indicate that the pre-impact motion features of control versus stroke individuals differ relative to the time of the fall impact itself. Stroke features related to falling were better detected further upstream from the fall impact, perhaps during the ambulation period leading up to a fall. However, this trend was not linear in either model. In some cases, for the stroke-trained model, increasing the lead time over 300 ms decreased the model performance. This finding suggests that investigating a variety of lead times may improve model performance evaluation, and could furthermore be beneficial to optimization of fall mitigation technologies (i.e. decreased allocation of expenses to maximally reducing the lead time of the device when not optimal). However, lead times should be optimized with the end user environmental domain in mind, especially during device validation. It may be of interest in future work to implement a time series classification structure in which previous movement states are used to track falls progressing over time, such as in a long short-term memory network. Such a model may benefit users requiring different windows of time and selection criteria to confidently pre-impact detect a fall at the earliest possible stage.

For this dataset, the direction of a fall seems to be of particular importance for model performance. Specifically, while the stroke-trained model is much better at detecting AP falls in post-stroke participants than the control-trained model, this improvement is less substantial when distinguishing falls to the side. This may be because stroke-related impairments do not affect the kinematics of all movements and functional activities equally. For example, Hollands et al. studied full-body kinematics of turning 180 degrees during the Timed Up and Go test [49] for patients following stroke compared to healthy age-matched controls. They found that while stroke survivors did take longer to turn, there were no significant kinematic differences demonstrated in turn performance or in axial segment coordination during turning between the two groups [50]. Extrapolating these results to our study, perhaps lateral falls for post-stroke individuals and healthy controls appear kinematically similar enough in IMU output that no significant improvements occur when training the model with stroke-specific data compared to control data. Nonetheless, the need for stroke-specific fall detection training relies on the hypothesis that there are significant and measurable differences in movement of individuals with stroke-related impairments, which we believe is demonstrated by the improved results for classification of AP falls. These nuances indicate a potential need for more research to distinguish which movements and falls are most effected kinematically by stroke-related impairments, so that stroke-specific model training can be minimized when control movement data is sufficient.

The current study design includes limitations. First, the fall detection model was developed based on a limited sample size. While this dataset is novel in its content of falls initiated from individuals with a history of stroke, future studies could expand upon the variety of data collected by using more participants with a wider range of demographics and impairment levels. A related confounding factor was that participants within the stroke cohort performed fewer falls and non-falls on average than control participants, due to considerations of safety and experiment duration. To reduce the effect of data imbalance between cohorts, the stroke model was trained on data from 19 post-stroke individuals, and the control model on data from 15 control individuals. For each tested stroke subject, this resulted in a stroke training data set with approximately 27.8% fewer non-fall events and 3.5% more fall events (only exceeding the control cohort in lateral fall count, in which there was no statistically significant difference in classification) as shown in Table 1. This training data arrangement was used while comparing the efficacy of the cohort-dependent trained models (Fig. 2) and evaluating the effect of non-fall type on fall detection (Table 2). While the training data sets as described provide a compromise between subject count and the quantity of trained samples between the two models, a leave-one-group-out schematic was additionally attempted to balance subject count in the analysis of stroke-related impairments (Fig. 3). In this case, the stroke training set had the same number of subjects, fewer falls, and fewer non-falls, but still has better or equal performance metrics. We believe this elevated performance, despite having fewer data to train with, is strong support for our central message: data from stroke subjects, especially of representative severity, is greatly beneficial for training pre-impact fall detection algorithms for the stroke population.

The method used to select participants with severe impairments in the stroke cohort is approximate and includes several assumptions. While many studies observe gait characteristics as indicators of post-stroke mobility deficits, such as gait speed and changes in trunk asymmetry and instability [48, 51–53]*,* these measures may not be the most indicative for risk of falling or movement severity. For the available data, it was not possible to assess whether greater range of motion about the lower trunk during gait was truly correlated to poor instability or was rather a by-product of compensatory mechanisms [53]. In addition, five of the stroke subjects could not be assessed for severity due to missing data. For true fall risk diagnosis in individuals with a history of stroke, numerous factors are taken into consideration and culminated to assess safe ambulation, including sensorimotor deficits, poor balance in a variety of contexts, visual and cognitive impairment, medication use, and impulsivity [5, 54]. While an increased quantity of the identified unstable ambulators in the test set changed performance as hypothesized, it remains uncertain whether performance change is attributed to true movement severity in this sub group, or rather to a specific movement pattern which our criterion selected for. In both cases, the results demonstrate that individuals with distinct movement kinematics will experience decreased user protection when not represented in the training set. It is recommended that future studies incorporate clinically validated assessment tools in addition to gait kinematics to label stroke impairment severity and thus, ensure that the final model accounts for individuals across the impairment spectrum. It may also be useful to consider the symptomatic cause of increased fall risk (e.g. hemiparesis vs loss of coordination) to further ensure inclusion of specific participant profiles. This stratification may allow for a more generalized, universal machine learning model for stroke impairment, or allow user input of an identified stroke presentation to fine tune parameters of a fall detection model accordingly.

While an aim of this study was to demonstrate the effect of non-falls complexity on model performance, more complex activities that occur in day-to-day living or which contribute to falling could have been included. Additionally, the models in this study were developed using the AdaBoost classification method, yet it is possible that other machine learning approaches which were not investigated for the purposes of this study might result in better performance. Rather than present the best classification model at this stage, this study aimed to demonstrate how training data composition can affect classification performance related to falls in stroke populations. While the statistical features used in these models did reveal statistically significant differences in the cohort-based model predictions, we aim to extract more clinically intuitive features with gold standard validation techniques in future work, as to better understand the kinematic differences between control and stroke populations. Finally, the airbag system was not deployed into a real-world setting, which is one of the next steps in our research. Conclusions made in regard to control- and stroke-trained models should be validated by the deployment of the model and prototype into the community for those individuals at risk of falling. Capturing real-fall data and testing model reliability and feasibility in both the community and clinical settings could strengthen the arguments made by this study.

## Conclusions

In summary, we present a sensor-driven wearable airbag technology for pre-detecting falls in individuals’ post-stroke. Results demonstrate the importance of developing fall mitigation technology which utilizes motion data specific to the stroke population. Even further, these results demonstrate that a “one-size-fits-all” pre-impact fall detection model based on healthy data may not extend to protecting other neurological, orthopedic, and neuromuscular conditions, including but not limited to Parkinson’s Disease, lower-limb amputation, and multiple sclerosis. Our insights could help researchers, clinicians, and companies to develop better fall detection models and advance fall mitigation technologies for population-specific individuals at high risk of falling. The use of such devices could help individuals after stroke and other conditions to reduce the risk of fractures and injuries and reduce their fear of falling, thus improving their overall health and their quality of life.

## Supplementary Information

**Additional file 1: Table S1.** Statistical features for pre-impact fall detection model.

**Additional file 2: Table S2.** Selection of unstable ambulators in the stroke cohort.

**Additional file 3: Fig. S1.** Modeling scheme to stratify stroke-related impairments.

**Additional file 4: Fig. S2.** Feature importance for a fully-trained stroke pre-impact fall detection model.

**Additional file 5.** Supplementary methods addressing detailed work and additional files Table S1, Table S2, Fig. S1, and Fig. S2.

## Data Availability

De-identified data are available from the corresponding author upon reasonable request.

## Abbreviations

AP: Anterior–posterior
ADL: Activity of daily living
AUC: Area under the receiver operating characteristic
IMU: Inertial measurement unit
