# Supplementary figures and images for: Wearable airbag technology and machine learned models to mitigate falls after stroke
Source: J Neuroeng Rehabil. 2022 Jun 17;19:60. doi: 10.1186/s12984-022-01040-4 (PMC9205156; doi:10.1186/s12984-022-01040-4)

## Slide 1
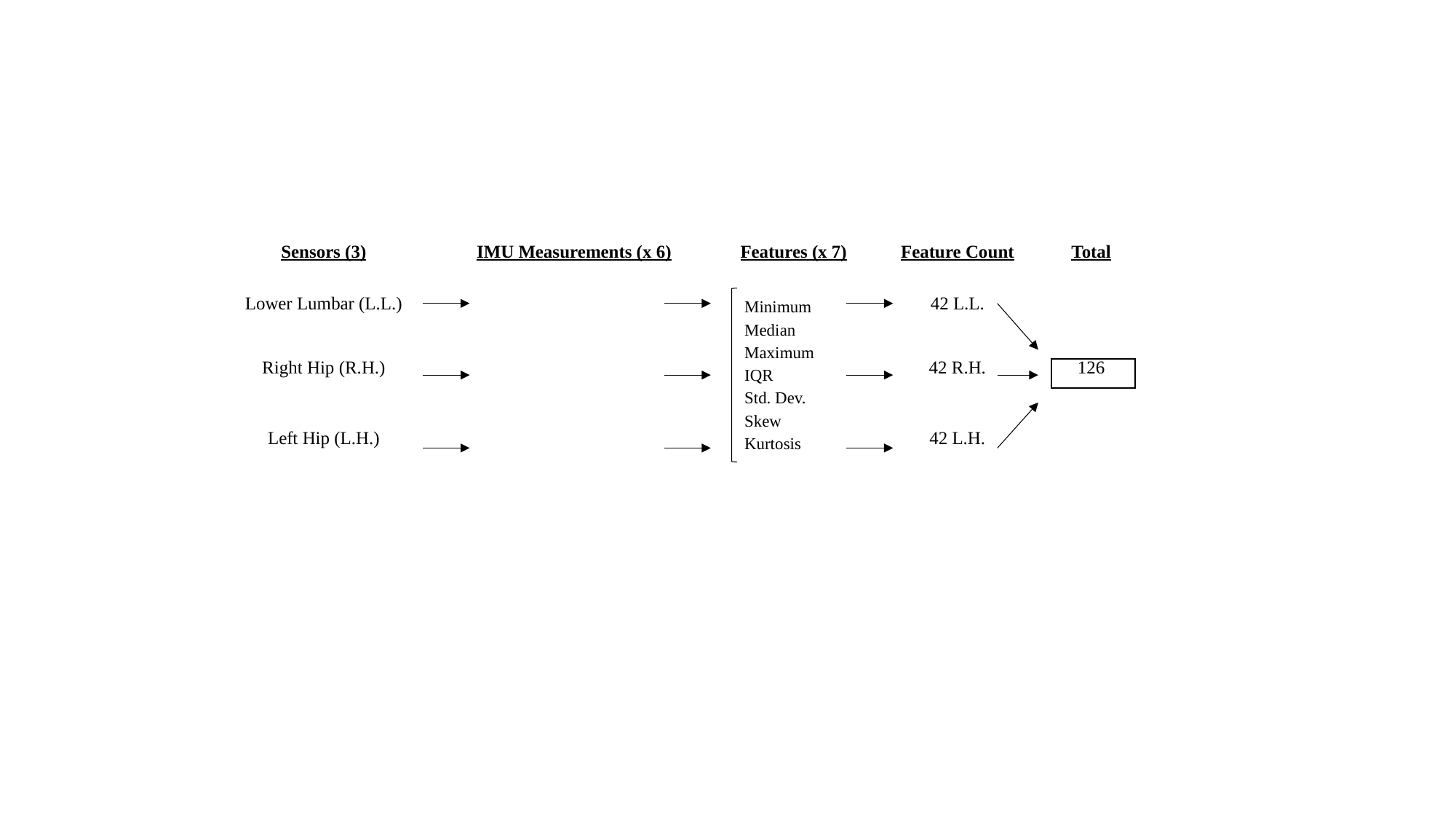

Supplement: Supplementary file 1 — Additional file 1: Table S1. Statistical features for pre-impact fall detection model. [file 12984_2022_1040_MOESM1_ESM.pptx]

## Slide 1
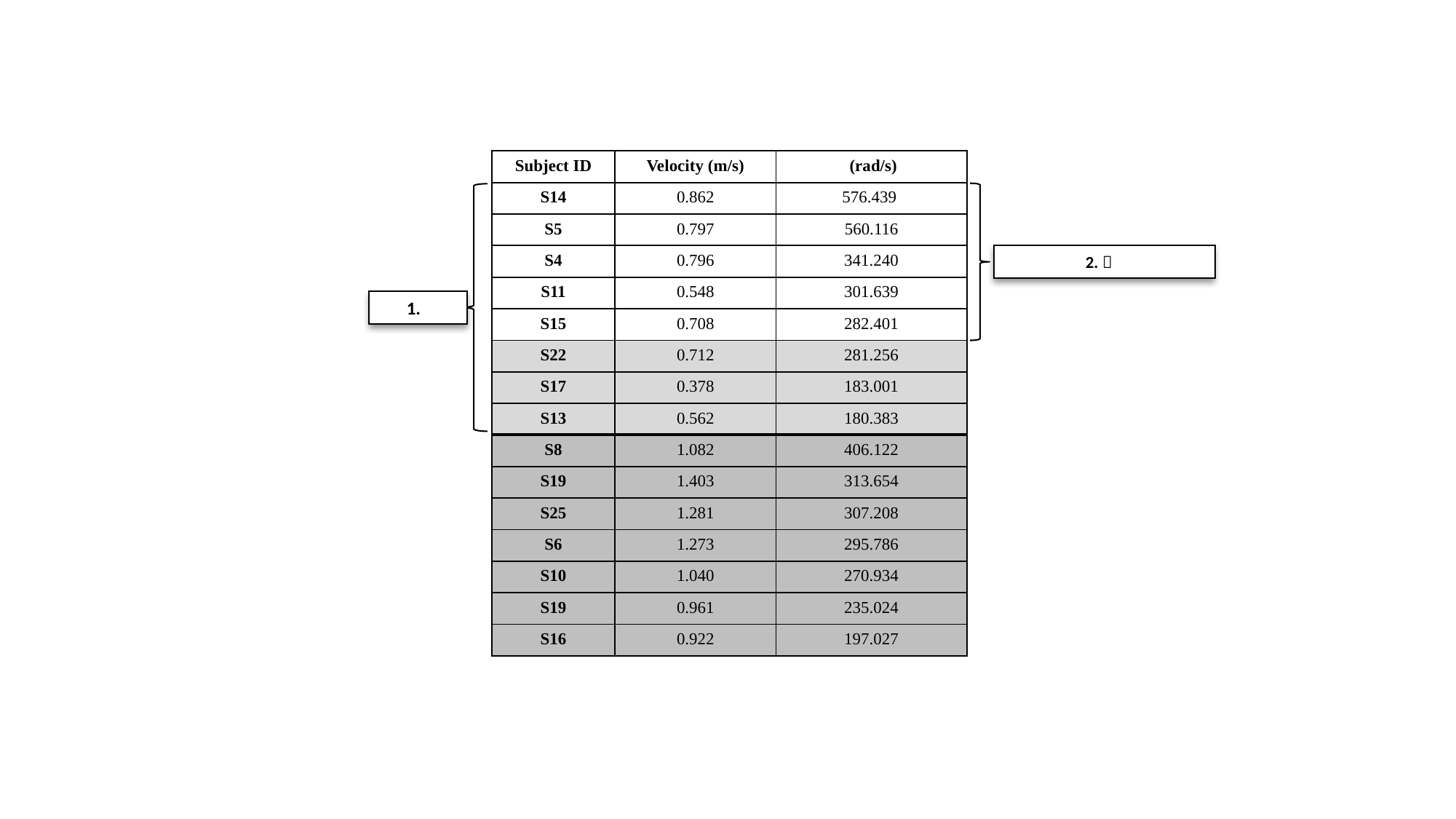

Supplement: Supplementary file 2 — Additional file 2: Table S2. Selection of unstable ambulators in the stroke cohort. [file 12984_2022_1040_MOESM2_ESM.pptx]
