# Supplementary material for: Wearable airbag technology and machine learned models to mitigate falls after stroke
Source: J Neuroeng Rehabil. 2022 Jun 17;19:60. doi: 10.1186/s12984-022-01040-4 (PMC9205156; doi:10.1186/s12984-022-01040-4)
Supplement: Supplementary file 5 — Additional file 5. Supplementary methods addressing detailed work and additional files Table S1, Table S2, Fig. S1, and Fig. S2. [file 12984_2022_1040_MOESM5_ESM.docx]

SUPPLEMENTARY MATERIALS

**Title: Wearable airbag technology and machine learned models to mitigate falls after stroke**

**Authors:** Olivia K. Botonis^1,*^, Yaar Harari^1,2,*^, Kyle R. Embry^1,2^, Chaithanya K. Mummidisetty^1^, David Riopelle^2,3^, Matt Giffhorn ^1^, Mark V. Albert^4^, Vallery Heike^5,6^, Arun Jayaraman^1,2,^ ^†^

**Affiliations:**

^1^ Max Nader Rehabilitation Technologies and Outcomes Lab, Shirley Ryan AbilityLab; Chicago, IL, USA.

^2^ Department of Physical Medicine and Rehabilitation, Northwestern University; Chicago, IL, USA.

^3^ Northwestern University Feinberg School of Medicine; Chicago, IL, USA.

^4^ Department of Computer Science and Engineering; Department of Biomedical Engineering, University of North Texas; Denton, TX, USA.

^5^ Department of BioMechanical Engineering, Delft University of Technology; Delft, The Netherlands.

^6^ Department of Rehabilitation Medicine, Erasmus MC; Rotterdam, The Netherlands.

↵* These authors should be considered co-first authors

↵† To whom correspondence should be addressed: Arun Jayaraman, PT, PhD,

E-mail: [a-jayaraman@northwestern.edu](mailto:a-jayaraman@northwestern.edu)

# SUPPLEMENTARY METHODS:

**Materials and Methods:**

As an exploratory analysis and extension to our study, we aimed to look beyond the binary presence or absence of stroke training data affecting pre-fall detection, and furthermore assess how severity of the stroke participants represented in the training phase impacts model performance. Specifically, we hypothesized that when testing on individuals with severe stroke impairments (i.e., high fall-risk), fall detection performance would decrease if individuals of a similar severity are not represented in the model training phase. Clinical evaluations are often used to assess severity of impairments and to predict fall risk in stroke rehabilitation *(1–5)*, yet several studies have claimed that the performance of fall risk prediction assessments are variable *(1, 6)* and limited upon validation *(7, 8)*. Recent studies suggest kinematic measures of instability during gait correlate to, or outperform, clinical assessments for instability and fall risk in the community *(8)*. Gait assessment is critical to understanding the risk factors contributing to fall events, as a majority of the falls in the community occur during ambulation *(9)*. To stratify stroke impairment severity of the participants, we utilized data collected for each participant during a standardized ten-meter walking trial while wearing the airbag IMUs. This walking data allows observation and comparison of general gait kinematics in a standardized format across participants, which may translate to the ambulatory motion leading up to a fall during fall pre-detection. We used IMU axes data from the sensor placed on the lower lumbar region to measure stroke-related motion and balance impairments during gait *(8, 10, 11)*.

Following suit of the aforementioned studies, we selected the following criteria to characterize subjects with greater instability and, consequently fall risk, relative to other participants within the cohort:

1. A gait speed below the median of the stroke population (0.84 m/s). Slower gait speed post stroke is often associated with more severe impairment and decreased likelihood of community ambulation *(12, 13)*.
2. A range of motion (ROM) in the upper quartile for the lower lumbar angular velocity about the x, y, or z axes (simulating trunk rotation, trunk flexion or extension, or increased or decreased weight shift, respectively). These exaggerated movements about a central point in the lower lumbar region indicate increased center of gravity displacement and instability during ambulation *(8, 14)*.

We labeled the participants of the stroke cohort whom meet this criterion as “unstable ambulators”, and tested how their presence in a sample population of test users affects pre-fall classification performance (**Fig.** **S1**). A compilation of test sets was constructed using a “leave-one-group-out” modeling scheme, where each group consists of five stroke subjects, thereby effectively balancing the number of training subjects (n = 15) between control and stroke models. Unique test groups, namely,

$C (n,k) = 15,504$ combinations for $n = 20$ stroke subjects, permutation size $k = 5$ ,

were labeled for the quantity of unstable ambulators contained in each test group’s elements. A final subset of these test groups was randomly selected in an attempt to balance the distribution among test groups. For test groups containing zero, one, two, or three unstable ambulators, 100 test groups were randomly selected per labeled quantity of unstable ambulators, and were input as test samples into both models. For test groups containing four or five unstable ambulators, all available samples were taken (i.e. 75 and 1 subgroup(s), respectively) and provided as input to both models.

**----- [Fig. S1] -----**

**SUPPLEMENTARY REFERENCES**

1. W. ME, H. NF, W. CD, G. R, Systematic review of risk prediction models for falls after stroke, *J. Epidemiol. Community Health* **70**, 513–519 (2016).

2. A. AH, A.-E. ES, A. S, S. B, Reliability, validity, and responsiveness of three scales for measuring balance in patients with chronic stroke, *BMC Neurol.* **18** (2018), doi:10.1186/S12883-018-1146-9.

3. D. PW, L. SM, K. J, Defining post-stroke recovery: implications for design and interpretation of drug trials, *Neuropharmacology* **39**, 835–841 (2000).

4. S. D, K. SJ, L. JM, T. JM, C. BL, L. DS, K. SE, Utility of the NIH Stroke Scale as a predictor of hospital disposition, *Stroke* **34**, 134–137 (2003).

5. A.-G. M, M.-A. JM, C.-S. JC, B.-S. S, P.-J. C, M.-F. A, de L.-R. ME, M.-S. AB, M.-B. AM, Instruments for assessing the risk of falls in acute hospitalized patients: a systematic review and meta-analysis, *BMC Health Serv. Res.* **13** (2013), doi:10.1186/1472-6963-13-122.

6. W. ME, G. R, B. F, W. D, H. JA, M. S, C. R, C. M, M. DJH, H. F, Validation of two risk-prediction models for recurrent falls in the first year after stroke: a prospective cohort study, *Age Ageing* **46**, 642–648 (2017).

7. C. M, T. J, B.-A. H, O. MW, Standardized Outcome Measures in Stroke Rehabilitation and Falls After Discharge: A Cohort Study, *PM R* **13**, 265–273 (2021).

8. M. Punt, S. M. Bruijn, H. Wittink, I. G. Van De Port, J. H. Van Dieën, Do clinical assessments, steady-state or daily-life gait characteristics predict falls in ambulatory chronic stroke survivors?, *J. Rehabil. Med.* **49**, 402–409 (2017).

9. V. Weerdesteyn, M. De Niet, H. J. R. Van Duijnhoven, A. C. H. Geurts, Falls in individuals with stroke, *J. Rehabil. Res. Dev.* **45**, 1195–1214 (2008).

10. F. Buganè, M. G. Benedetti, V. D’Angeli, A. Leardini, Estimation of pelvis kinematics in level walking based on a single inertial sensor positioned close to the sacrum: validation on healthy subjects with stereophotogrammetric system, *Biomed. Eng. Online* **13** (2014), doi:10.1186/1475-925X-13-146.

11. M. K. O’brien, M. D. Hidalgo-Araya, C. K. Mummidisetty, H. Vallery, R. Ghaffari, J. A. Rogers, R. Lieber, A. Jayaraman, Augmenting clinical outcome measures of gait and balance with a single inertial sensor in age-ranged healthy adults, *Sensors (Switzerland)* **19** (2019), doi:10.3390/s19204537.

12. P. J, G. M, G. JK, M. SJ, Classification of walking handicap in the stroke population, *Stroke* **26**, 982–989 (1995).

13. G. D. Fulk, Y. He, P. Boyne, K. Dunning, Predicting Home and Community Walking Activity Poststroke, *Stroke* **48**, 406–411 (2017).

14. M. Patel, A. Pavic, V. A. Goodwin, Wearable inertial sensors to measure gait and posture characteristic differences in older adult fallers and non-fallers: A scoping review, *Gait Posture* **76**, 110–121 (2020).

**
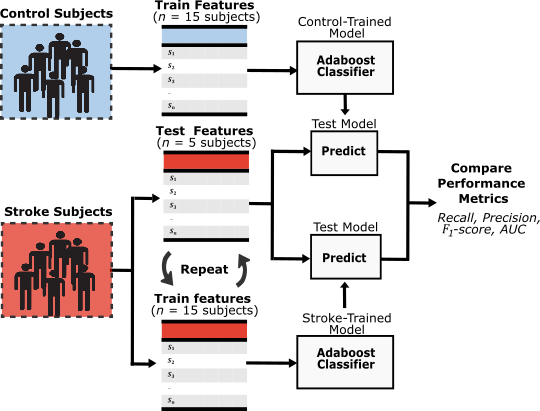
Figures**

**Fig. S1. Modeling Scheme to Stratify Stroke-Related Impairments.** Modeling scheme to split train and test groups for control- and stroke-trained models in the stroke-related impairment analysis. Each test group of features (*n* = five stroke subjects) were set aside for testing. The control model was trained on data of all control subjects (*n* = 15), while the stroke model was trained on data of all stroke subjects not in the held out test group (*n* = 15), thereby balancing the number of subjects trained upon in each model. This process was repeated for all randomly selected test groups, and average performance metrics were reported for each model (stratified by the labeled quantity of unstable ambulators- see corresponding results in **Figure 3**).
